# Hydrogen and Cushion Gas Adsorption–Desorption Dynamics on Clay Minerals

**DOI:** 10.1021/acsami.4c12931

**Published:** 2024-09-26

**Authors:** Qian Zhang, Mohammad Masoudi, Lingjie Sun, Lunxiang Zhang, Lei Yang, Yongchen Song, Aliakbar Hassanpouryouzband

**Affiliations:** † Key Laboratory of Ocean Energy Utilization and Energy Conservation of the Ministry of Education, Dalian University of Technology, Dalian 116024, China; ‡ Department of Geosciences, University of Oslo, P. O. Box 1047 Blindern, 0316 Oslo, Norway; § Applied Geoscience Department, SINTEF Industry, 7465 Trondheim, Norway; ∥ Ningbo Institute of Dalian University of Technology, No.26 Yucai Road, Jiangbei District, Ningbo 10315016, China; ⊥ School of Geosciences, University of Edinburgh, Grant Institute, West Main Road, Edinburgh EH9 3FE, United Kingdom

**Keywords:** geological hydrogen storage, natural hydrogen, isothermal adsorption, physisorption, clay minerals, hysteresis, desorption

## Abstract

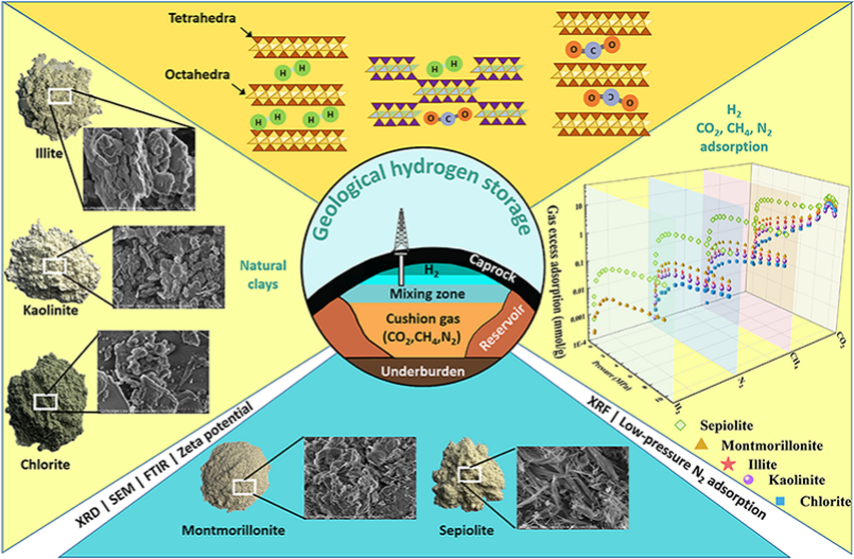

Transitioning toward a hydrogen (H_2_)-centric energy paradigm necessitates understanding the adsorption properties of clay minerals, essential constituents of reservoirs and caprocks, for efficient geological H_2_ storage. This study examines the adsorption characteristics of H_2_ on various clay minerals (montmorillonite, illite, chlorite, kaolinite, and sepiolite) at different temperatures and the adsorption of cushion gases (N_2_, CH_4_, and CO_2_) under reservoir conditions (313.15 K, up to 10 MPa). The results indicate that sepiolite demonstrates superior adsorption capacity under all tested conditions, surpassing montmorillonite by over 12 times at 313.15 K for H_2_. Illite, chlorite, and kaolinite exhibit negligible H_2_ adsorption. Thermodynamic analysis reveals that H_2_ adsorption on clay minerals is a nonspontaneous and exothermic physisorption process. H_2_ loss due to adsorption hysteresis in montmorillonite and sepiolite is 42.19 and 3.56%, respectively. Sepiolite may exhibit more predictable and stable sorption properties under repeated pressure variations. The H_2_ adsorption capacity of montmorillonite and sepiolite is merely 0.4 and 4.5% of that of CO_2_, respectively. This study provides valuable insights for selecting clay minerals and cushion gases for efficient geological H_2_ storage and natural hydrogen exploration.

## 1 Introduction

The global energy landscape is undergoing a significant transformation, with an increasing emphasis on sustainable and renewable energy sources.^1,2^ Hydrogen (H_2_) is emerging as a key component of this transition due to its high energy density and potential for reducing greenhouse gas emissions.^3,4^ One of the critical challenges in leveraging hydrogen as a clean energy carrier is the efficient and safe storage of hydrogen, particularly in large-scale geological formations.^5−8^

Clay minerals, prevalent in many geological formations, are of particular interest for hydrogen storage due to their high surface area, porosity, and unique structural properties.^9,10^ Among the various clay minerals, montmorillonite (Mt), Illite (Il), chlorite (Chl), sepiolite (Sep), and kaolinite (Kaol) are commonly found in subsurface environments.^11^ These minerals exhibit diverse adsorption behaviors, making them suitable candidates for detailed investigation.

Understanding the adsorption characteristics of these clay minerals is crucial for optimizing hydrogen storage systems. Adsorption isotherms, such as those described by the Langmuir model, provide valuable insights into the adsorption capacity and behavior of gases on solid surfaces. Early research on H_2_ adsorption by clay minerals primarily focused on their use as adsorbents for surface gas storage due to their low cost, natural abundance, and high stability. These results demonstrate the feasibility of H_2_ adsorption on clay mineral surfaces.^12,13^ Furthermore, the H_2_ adsorption capacity has been enhanced through modifications of clay minerals, such as intercalation with oligocations,^12^ metal doping,^13,14^ and acid treatment.^14,15^ However, the use of modified or synthetic non-natural clay minerals and/or the nonreservoir conditions of low temperature and low pressure employed in these studies may complicate subsequent evaluations for large-scale geological H_2_ storage. Due to the urgent need for an energy transition, the adsorption of H_2_ by clay minerals under reservoir conditions has garnered significant attention in the context of geological H_2_ storage in recent years. As shown by Truche et al.,^16^ the presence of clay components such as Illite, chlorite, and kaolinite in the rock mineralogy can lead to trapping and complicate the storage process. Ziemiański et al.^17^ performed H_2_ adsorption experiments on various cationic forms of montmorillonite, beidellite, and Illite at temperatures of 298, 323, and 343 K under pressures up to 15 MPa. Their findings demonstrated that H_2_ adsorption in clay minerals is strongly dependent on mineral texture. Wolff-Boenisch et al.^18^ conducted H_2_ adsorption experiments on natural montmorillonite at a pressure of 5 MPa and temperatures of 77, 195, and 303 K. Their findings revealed that the interaction between H_2_ and montmorillonite was weak. They suggested that deeper reservoirs could enhance storage capacity and reduce H_2_ loss through clay adsorption in the caprock. Wang et al.^19^ investigated H_2_ adsorption behavior on different clay minerals (montmorillonite, chlorite, sepiolite, palygorskite, kaolinite, and Illite) at 273, 298, 318, and 348 K, and pressures up to 18 MPa. Their results showed that H_2_ adsorption is primarily controlled by the pore structure, with pores below 30 nm being more adsorptive for H_2_. To summarize, there is still a lack of sufficient experimental adsorption isotherm data in this field, particularly for adsorption on pure clay minerals under reservoir conditions. Additionally, the available data on H_2_ adsorption on clays is quite scattered. Owing to variations in experimental conditions (covering different ranges of temperature and pressure) and the intrinsic properties of clay minerals, the results of different studies may be incomparable.

Furthermore, the selection of appropriate cushion gases, such as CO_2_, N_2_, and CH_4_, plays a pivotal role in enhancing the storage capacity and stability of hydrogen in geological formations.^20^ The selection of cushion gases necessitates the evaluation of various factors, and extensive research has been carried out to investigate these factors through experimental^21−23^ and simulation^24−29^ methods. Current studies mainly focus on intrinsic physical properties of gases like density,^30,26,28^ viscosity,^24^ diffusivity,^25,28,29^ and solubility,^27,31^ as well as interactions with the reservoir, including aspects such as interfacial tension,^32,26^ wettability,^22,26^ and adsorption characteristics.^21,23,33^ Among these aspects, the selection of cushion gases from an adsorption perspective is less thoroughly explored. Existing research on the adsorption behavior of cushion gases has predominantly focused on coal,^21,23^ and shale formations.^33^ In contrast, studies on the adsorption capacity of various clay minerals have generally yet to consider the adsorption capacity of cushion gases on these different clay minerals under consistent conditions.

Operational integrity during multiple hydrogen removal and injection cycles is a key consideration for the viability of underground hydrogen storage solutions. Repeated cycling can impact the storage efficiency and overall performance of the storage material. This evaluation specifically examines the presence of hysteresis, a phenomenon where the adsorption and desorption paths do not coincide, leading to potential inefficiencies in storage capacity. However, no previous studies investigated that.

This study aims to systematically investigate the hydrogen adsorption capacity of various clay minerals using high-pressure adsorption isotherms and the Langmuir model. By examining the structural and compositional characteristics of these minerals, this research seeks to provide insights into their suitability for large-scale hydrogen storage. Moreover, the implications of these findings for reservoir site selection and cushion gas strategies will be discussed, offering a comprehensive perspective on optimizing geological hydrogen storage.

## 2 Experimental Methodology

### 2.1 Materials

The gases used as adsorbate were hydrogen (H_2_), carbon dioxide (CO_2_), methane (CH_4_), nitrogen (N_2_), and helium (He), all with a purity of 99.99% purity (Dalian Special Gases Co., Ltd., China).

The adsorbents used included Ca-montmorillonite (Ca-Mt), Illite (Il), chlorite (Chl), sepiolite (Sep), and kaolinite (Kaol), sourced from Shanlinshiyu Mineral Products Co., Ltd., China. To accurately assess H_2_ adsorption on natural clay minerals, we selected clay minerals of high purity with minimal nonclay impurities as our research material. The samples were ground and sieved to obtain homogeneous particle sizes finer than 325 mesh (<45 μm).

### 2.2 Characterization of Clay

The mineralogical composition was analyzed using X-ray diffraction (XRD) with a scanning range of 3–70° (2θ) at a rate of 8°/min, employing Cu Kα radiation at 40 kV and 40 mA (D8 Advance, Bruker, Germany). The chemical composition of the samples was determined using X-ray fluorescence (XRF) through quantitative analyses (Axios, PANalytical, Netherlands).

The specific surface area and pore size distribution of the samples were calculated using the Brunauer–Emmett–Teller (BET) method^34^ and density functional theory (DFT) model,^35^ respectively, based on low-pressure nitrogen adsorption (LP-N_2_GA) experiments conducted at 77 K (Micromeritics ASAP 2425 Adsorption Analyzer). The surface functional groups of samples were identified using a Fourier Transform Infrared Spectrometer (FTIR, iS50, Thermo Fisher). The surface morphological features of the samples were observed using a scanning electron microscope (SEM) operated at a voltage of 3 kV for each measurement (SU5000, Hitachi, Japan). The surface zeta potential of the samples was measured using a surface zeta potential analyzer (Nano-ZS90, Malvern, UK).

### 2.3 High-Pressure Gas Adsorption Measurements

#### 2.3.1 Gas Adsorption Isotherm Tests

To determine the high-pressure gas adsorption of samples, we employed the volumetric method using an automatic high-pressure gas adsorption instrument equipped with a temperature-controlled circulator (Isorb HP2, Quantachrome). Prior to the actual analysis, the clay minerals were dried at 373.15 K in an oven for over 10 h. To eliminate moisture and any trace pollutants adsorbed during the installation of the adsorption cell, the samples were degassed at 473.15 K under constant vacuum for 6 h. The degassing temperature was selected based on the prior studies, which indicate it effectively removes all bound and capillary water adsorbed on the clays without inducing any irreversible changes to their microstructures.^36−38^

Helium, as a nonadsorptive reference gas, was used to check for leaks. The dead volume of the sample cell was assessed across various temperatures employing the He expansion technique. The gas adsorption capacity was derived from the pressure measurements before and after expansion, utilizing a suitable equation of state. Specifically, the mBWR-Jacobsen equation was used for H_2_, the Helmholtz equation for N_2_ and CH_4_, and the Peng–Robinson equation for CO_2_.

The experimental conditions of this study encompassed a pressure range of 0 to 10 MPa and spanned five different temperatures: 273.15, 283.15, 293.15, 303.15, and 323.15 K. Hydrogen adsorption tests were conducted on all clay samples at 273.15 and 283.15 K. At 293.15, 303.15, and 323.15 K, these tests were limited to montmorillonite and sepiolite. At 313.15 K, adsorption tests for hydrogen, nitrogen, methane, and carbon dioxide were performed on all samples. A comprehensive summary of the experimental conditions is provided in Table S1 of the Supporting Information.

#### 2.3.2 H_2_ Adsorption–Desorption Tests: Assessing Hysteresis and Injection/Production Cycles

We explored the adsorption–desorption behavior of montmorillonite and sepiolite in the context of their potential use in subsurface hydrogen storage. This evaluation is crucial, as operational integrity during multiple hydrogen removal and injection cycles is a key consideration for the viability of underground hydrogen storage solutions. The study specifically examines the presence of hysteresis and its impact on storage efficiency. The procedure of the tests is the same as the other high-pressure tests, but after the adsorption–desorption experiment, the next adsorption–desorption experiment was started directly without degassing the samples in the sample tubes. This process was repeated 10 times.

### 2.4 Langmuir Model for Quantifying H_2_ Adsorption Capacity in Clay

Adsorption isotherm modeling is instrumental in elucidating the fundamental adsorption properties of materials. The Langmuir isotherm model, appreciated for its straightforward parameters and relevance to geological contexts,^23,39,40^ stands out as a frequently utilized approach in estimating the amount of adsorbed gases in reservoirs. Therefore, in this study, we applied the Langmuir model to fit the measured isotherms premising on the theory of monolayer H_2_ adsorption. The Langmuir adsorption model is represented by eq 1
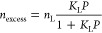
1Where *n*_excess_ (mmol/g) indicates the excess adsorbed amounts at a typical gas pressure of *P* (MPa); *n*_L_ (mmol/g) is the maximum Langmuir capacity, which is the adsorbed amount at full occupancy of the Langmuir monolayer; and *K*_L_ (MPa^–1^) denotes the Langmuir constant, an essential parameter for evaluating the feasibility of gas desorption under reservoir pressure. The Langmuir pressure constant (*P*_L_, in MPa), expressed as the reciprocal of the Langmuir constant, corresponds to the pressure at which the gas storage capacity equals one-half of the maximum gas adsorption capacity, i.e., *P* = *P*_L_ when *n* = *n*_L_/2.

The temperature dependence of the adsorption process can be described by the variation of the Langmuir constant (*K*_L_) with temperature, as expressed by the thermodynamic parameters in eq 2
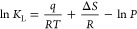
2Where *q* represents the heat of adsorption (kJ/mol), which is equal in magnitude to the enthalpy of adsorption Δ*H* but with a negative sign (*q* = –Δ*H*); *T* is temperature (K); *P* is the standard atmospheric pressure (0.1 MPa); Δ*S* is standard entropy of adsorption (J/mol/K), and *R* is the gas constant (8.3145 J/mol/K). The *q* and Δ*S* parameters are determined from the slope and the *y*-axis intercept, respectively, based on the plot of ln *K*_L_ versus 1/*T*.

Thermodynamic properties offer valuable insights for a better understanding of the spontaneity of an adsorption mechanism. Adsorption is a spontaneous process if it is characterized by a decrease in the total free energy of the system.^41^ The Gibbs free energy, Δ*G* (kJ/mol), values are determined using any of eqs 3 or 4
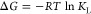
3
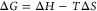
4

## 3 Results and Discussion

### 3.1 Structural Characterization of Clay Minerals

**Figure 1:**
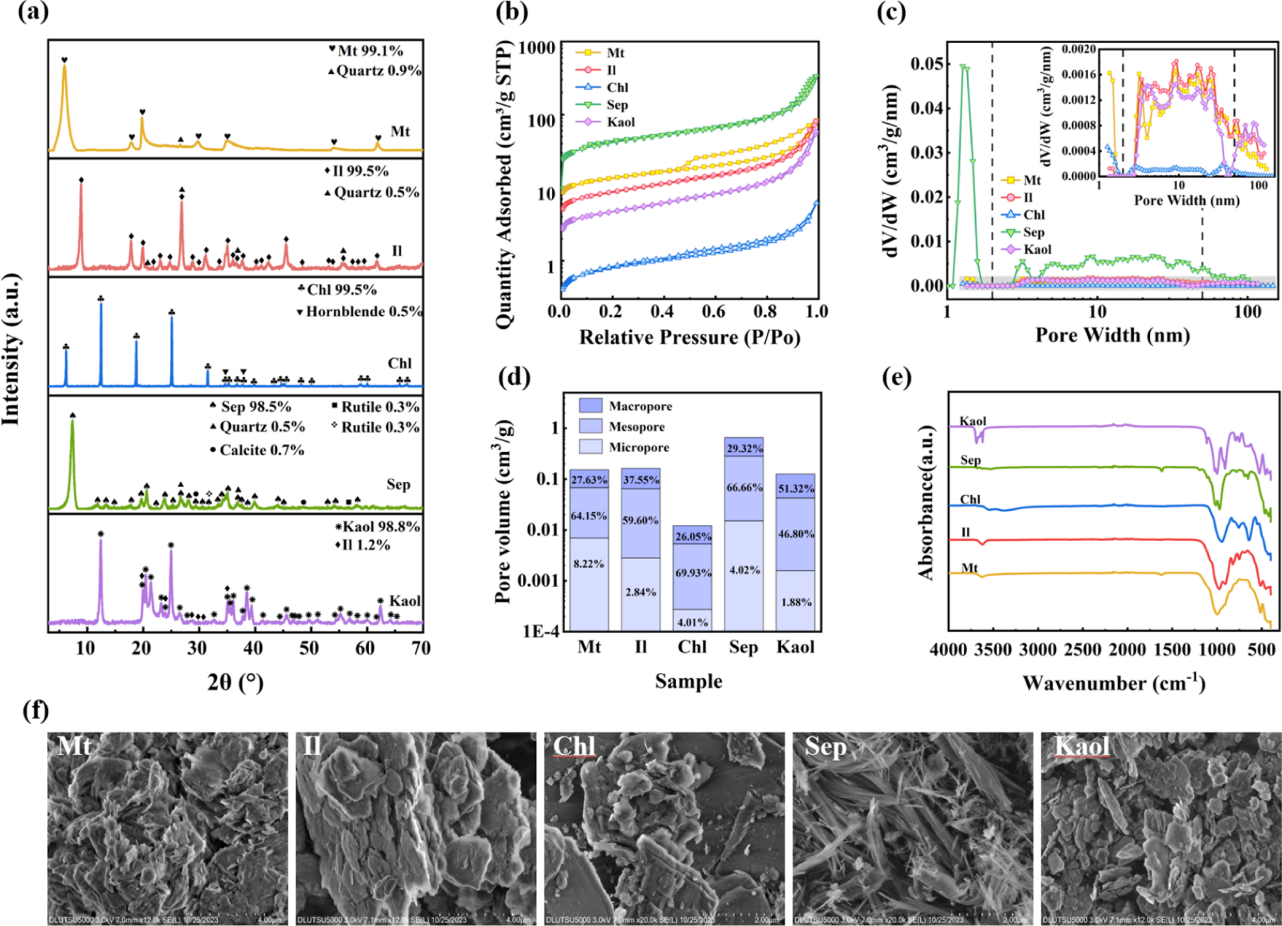
Characterization results of five samples: (a) XRD patterns, showing the samples primarily composed of clay minerals with a purity of over 98.5%. (b) N2 adsorption–desorption isotherms, displaying IUPAC type IV isotherms with H2 hysteresis loops for Mt and H3 hysteresis loops for the other samples. (c) Pore size distribution (PSD) following the IUPAC pore classification: micropores (< 2 nm diameter), mesopores (between 2 and 50 nm), and macropores (> 50 nm). (d) Micropore, mesopore, macropore, and total pore volume. (e) FTIR spectra. (f) Representative SEM images: Mt displays a cauliflower-like structure, Il shows irregular, scale-like aggregates, Chl forms rosette-like structures, Sep exhibits a fibrous structure, and Kaol presents an irregular hexagonal plate morphology.

To understand the nature of gas adsorption on clay minerals, we characterized the samples using XRD, XRF, LP-N_2_GA, FTIR, SEM, and surface zeta potential analyses. The outcomes of our analysis are depicted in Figure 1 and discussed in detail below.

The XRD patterns of all samples are presented in Figure 1a, revealing that they are primarily composed of clay minerals with minor impurities, including quartz, calcite, and hornblende. The samples exhibit a remarkable purity of over 98.5%, as confirmed by XRD analysis. Additionally, to improve quantitative accuracy, we integrated elemental analysis data obtained from XRF tests. The results for these five clay minerals are presented in Table S2. Major quantities of SiO_2_ and Al_2_O_3_ are present in all the clays. In addition to these, minor amounts of Fe_2_O_3_, MgO, CaO, TiO_2,_ K_2_O, P_2_O_5,_ MnO, and Na_2_O are also present as metallic oxides.

Nitrogen adsorption–desorption isotherms were used to explore hysteresis loops and elucidate sample pore shapes. They also provide insight into pore volume, specific surface area, and pore size distribution, which are critical parameters for assessing gas adsorption capacity. As shown in Figure 1b, the nitrogen adsorption isotherms of all five clay samples exhibit IUPAC type IV isotherms with type II adsorption branches, indicating the presence of well-developed mesopores (2–50 nm) in the studied samples. Mt displays a hysteresis loop of type H2, indicating inkbottle-shaped pores or polymorphic pores are the predominant pores, followed by slit-shaped pores and/or macropores. In contrast, Il, Chl, Sep, and Kaol samples mainly show a hysteresis loop of type H3, suggesting the presence of cracks, wedge structures, and/or layered capillary pores formed between plate-like particles. It is noteworthy that samples exhibiting type H2 hysteresis loops contain more micropores (<2 nm) compared to those with type H3 hysteresis loops.^42^

Based on DFT methods, the samples were analyzed. As illustrated in Figure 1c, Mt, Chl, and Sep exhibit a bimodal distribution, with modes of around less than 1.6 and 2.9–43 nm. Mt, Il, and Kaol display similar PSD in the mesoporous range. Note that the relative contributions of micropores, mesopores, and macropores to the total pore volume are presented as percentages in the corresponding histograms (Figure 1d).

The pore volumes and surface areas calculated from the LP-N_2_GA data are listed in Table 1. The specific surface area (SSA) measured by BET method can be ranked as follows: Sep ≫ Mt > Il > Kaol > Chl. The volume of micropores for the studied samples follows a similar order (Table 1 and Figure 1d). Solid material adsorption capacity is typically proportional to the SSA, as a larger SSA provides more binding sites for gas adsorption. Sep showed a significantly larger pore volume than the other four samples, resulting in a larger SSA.

**Table 1:** Pore Structure Characteristics of Studied Samples

samples	*S*_BET_ (m^2^/g)	*V*_Total_ (cm^3^/g)	*V*_micropore_ (cm^3^/g)	*V*_mesopore_ (cm^3^/g)	*V*_macropore_ (cm^3^/g)
Sep	149.9377	0.3796	0.0153	0.2531	0.1113
Mt	47.9975	0.0851	0.0070	0.0546	0.0235
Il	31.0006	0.0994	0.0028	0.0592	0.0373
Kaol	16.5665	0.0846	0.0016	0.0396	0.0434
Chl	2.9577	0.0069	0.0003	0.0048	0.0018

FTIR spectroscopy was employed to analyze the composition, structure, and surface characteristics of the samples, as well as to gather information about water (H_2_O) and hydroxyl groups (OH). The valuable “fingerprint” absorptions in clay mineral characterization primarily stem from the stretching and bending vibration bands of Si–O, Al–O, and O–H.^43^

The FTIR spectra of clay minerals are shown in Figure 1e. Stretching vibrations of OH groups appear in the range of 3650–3300 cm^–1^. The OH vibrational absorption peaks are observed at 3621 cm^–1^ for Mt and Il, at 3538 and 3391 cm^–1^ for Chl, at 3532 cm^–1^ for Sep, and 3695, 3652, and 3619 cm^–1^ for Kaol. Despite heating and drying the samples prior to analysis, the clear bands at 1620 cm^–1^ for Mt and 1613 cm^–1^ for Sep could be attributed to the deformation vibrations of OH-adsorbed water. The absorption peaks in the range of 1200–400 cm^–1^ correspond to the bending vibration of the hydroxyl group. Additionally, Si–O stretching vibrations typically occur at 1200–800 cm^–1^, while the related bending bands are found in the range of 600–400 cm^–1^ in the FTIR spectra of minerals.

Under SEM observation, Mt displays a cauliflower-like structure with curled edges and densely packed particles. These structures are tightly stacked but display interlayer spaces. Il shows irregular, scale-like aggregates with compact accumulation resulting from significant compaction, characterized by larger particle sizes and minimal interlayer porosity. Chl forms rosette-like structures, with particles that are the smallest and most dispersed, showing incomplete development, low porosity, and sparse pores. Sep exhibits a fibrous structure, with elongated particles forming a highly porous, open network. The fibers are loosely arranged, with distinct voids between them. Both Mt and Sep display abundant micropores and mesopores, exposing more active sites and enhancing gas adsorption. Kaol presents an irregular hexagonal plate morphology and partially forms a book-like structure agglomerated structure. Kaol also exhibits significant porosity, though its pores are relatively larger, predominantly consisting of mesopores. These observations are consistent with the PSD analysis results (Figure 1f).

The zeta potentials of Mt, Il, Chl, Sep, and Kaol were measured as −13.3, −33.4, −27.3, −20.8, and −21.1 mV, respectively. These measurements were conducted at a pH of 9.0 and a temperature of 298.15 K using deionized water.

### 3.2 H_2_ Adsorption Capacity of Clay Minerals

#### 3.2.1 H_2_ Adsorption Isotherm of Clay Minerals

**Figure 2:**
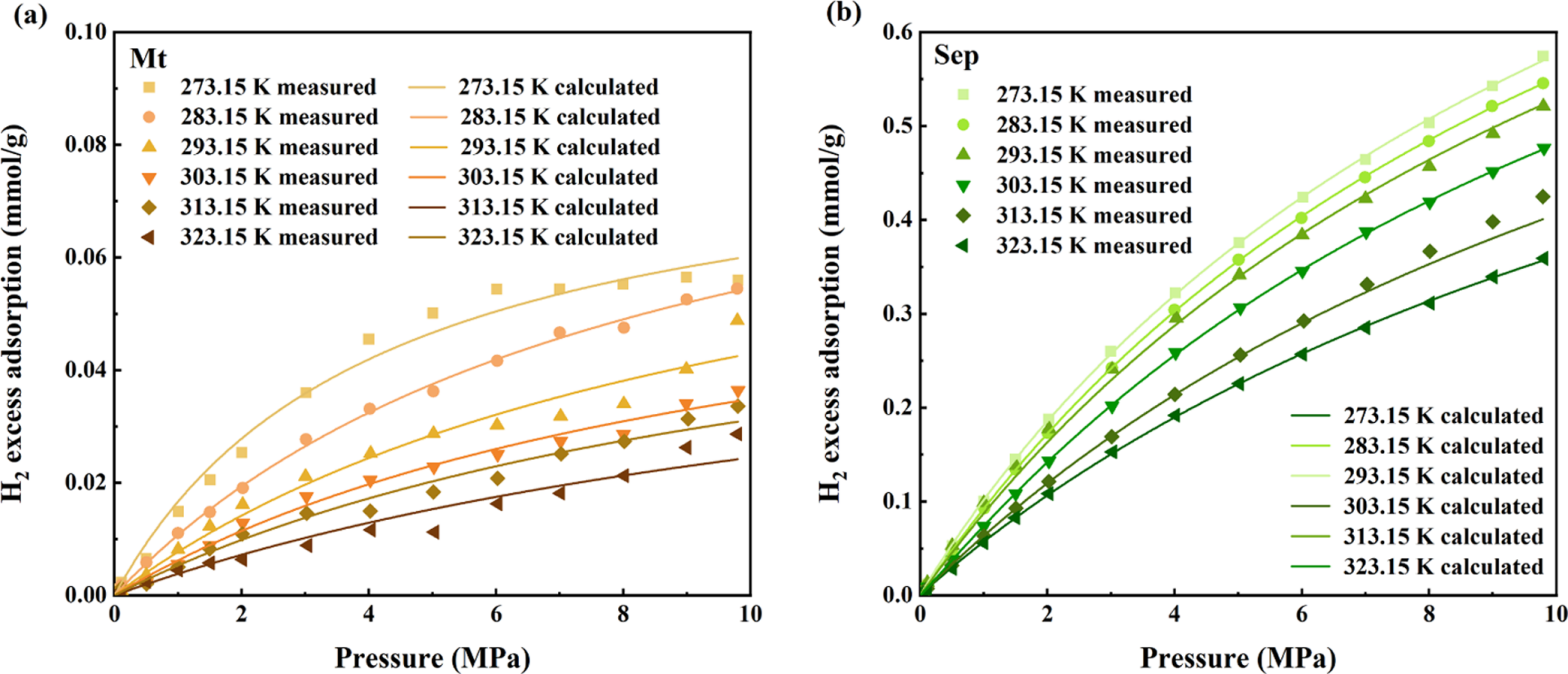
High-pressure H_2_ adsorption isotherms for (a) Montmorillonite (Mt) and (b) Sepiolite (Sep). The discrete data points indicate the experimental measurements, while the continuous lines depict the quantities modeled via the Langmuir isotherm equation.

H_2_ adsorption experiments were conducted under pressures up to 10 MPa and at various temperatures. As demonstrated in Figure 2, significant differences in the H_2_ adsorption capacity were observed between montmorillonite and sepiolite, with sepiolite exhibiting the highest capacity for H_2_ adsorption. In contrast, Illite, chlorite, and kaolinite displayed negligible adsorption capabilities.

The higher adsorption potential of Sep and Mt can be attributed to several intrinsic properties. As shown in the SEM images (Figure 1f), sepiolite has a unique fibrous morphology that contributes to its high porosity and specific surface area. This porous structure creates numerous adsorption sites, enhancing its capacity to adsorb hydrogen. The mesopores and micropores within Sep are ideally sized to trap hydrogen molecules more efficiently compared to other minerals.

Mt, on the other hand, has a 2:1 layer structure, consisting of two tetrahedral sheets sandwiching an octahedral sheet. This structure allows for greater interlayer spacing, which can expand to accommodate water molecules and gas adsorbates, thereby enhancing its adsorption capacity. As displayed in Table 1 and Figure 1d, both sepiolite and montmorillonite have larger specific surface areas, higher pore volumes, and greater micropore volumes. A larger surface area provides more active sites for hydrogen adsorption, thus increasing the overall adsorption capacity.

Additionally, FTIR results suggest a strong presence of OH groups in Sep and Mt, correlating with their high H_2_ adsorption capacities due to highly effective active sites and structural advantages. However, despite having OH vibrations, Il, Ch, and Kaol show negligible adsorption capacities. This suggests that their structural properties and lower surface areas overshadow the potential benefits indicated by FTIR alone.

Interestingly, the results of zeta potential do not correlate with H_2_ adsorption. While zeta potential is a crucial parameter, it does not solely dictate adsorption capacity. The higher adsorption capacity of Mt, despite its less negative zeta potential, can largely be attributed to its structural advantages and other surface properties that significantly aid in gas adsorption. Similarly, Sep’s adsorption efficiency is aligned with its structural attributes and not just its surface charge. These values reflect the multifaceted nature of adsorption phenomena, where multiple factors interplay.

#### 3.2.2 Fitted Parameters of the Langmuir-Based Excess Adsorption Model

To further assess H_2_ adsorption across a range of temperatures (273.15, 283.15, 293.15, 303.15, 313.15, and 323.15 K), we selected Mt and Sep due to their high H_2_ storage capacities among the five clay minerals studied. H_2_ adsorption capacity clearly varies as a function of temperature and pressure (Figure 2).

**Figure 3:**
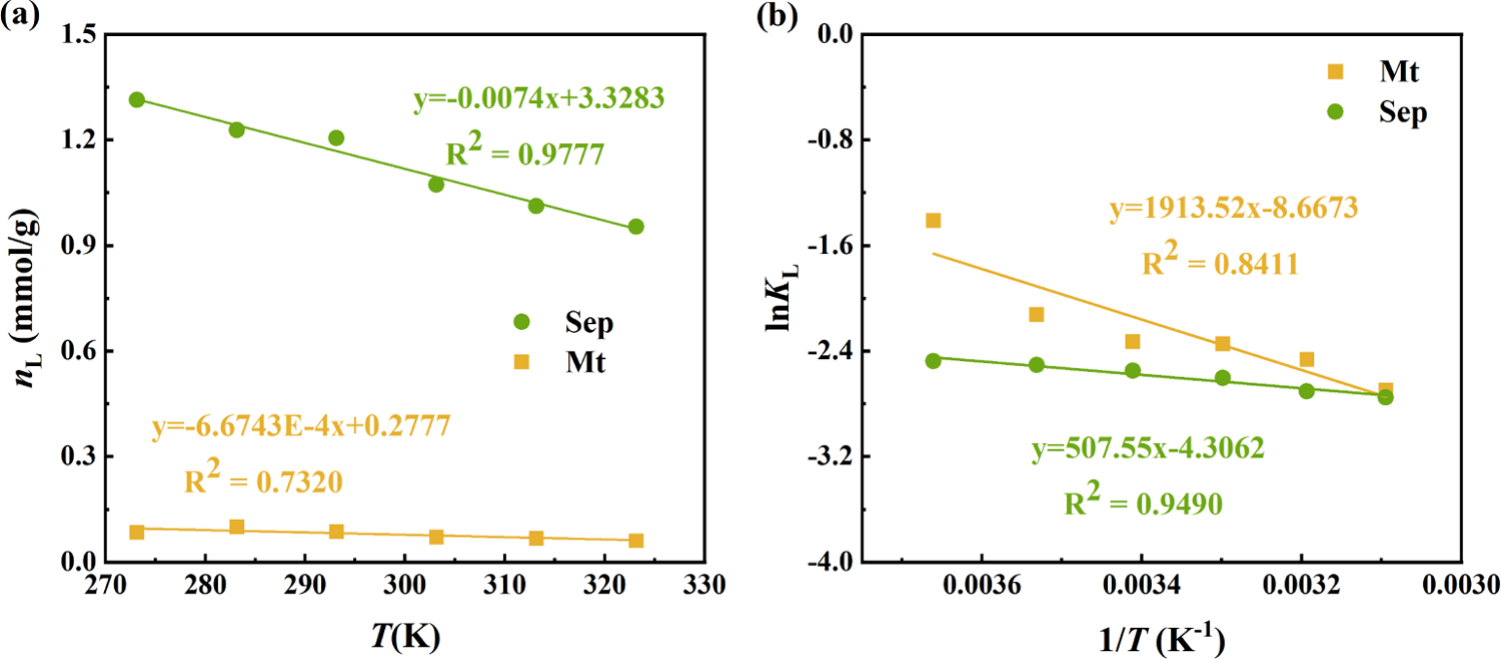
(a) Plots of the *n*_L_ versus temperature for two clay samples. (b) The plot of natural logarithms of the *K*_L_ versus the reciprocal of temperature (1/*T*) for two clay samples.

We applied a least-squares fit to our experimentally measured H_2_ adsorption isotherms using the Langmuir function (eq 1) to calculate the maximum Langmuir adsorption capacity (*n*_L_) and the Langmuir constant (*K*_L_). As illustrated in Figure 3 and Table S3, the Langmuir-based excess adsorption function (eq 1) aligns closely with the experimental excess adsorption isotherms, validating the suitability of the Langmuir model in describing H_2_ adsorption on clay minerals. Sep exhibits notably higher *n*_L_ and *P*_L_ values compared to Mt. Additionally, a decrease in *K*_L_ values for both Mt and Sep is observed with increasing temperature.

Temperature significantly impacts the adsorption capacity of various clay minerals. As shown in Figure 2, the adsorption capacity of H_2_ decreases significantly with increasing temperature. As the temperature rises, the thermal motion of H_2_ molecules intensifies, which increases the kinetic energy of the H_2_ molecules and reducing the strength of the binding forces between the H_2_ and clay minerals. This process impedes H_2_ adsorption and promotes desorption, resulting in a reduction in the adsorption capacity of clay minerals. Consequently, the adsorption process is more effective at lower temperatures. Additionally, the Langmuir maximum adsorption capacity (*n*_L_) demonstrates a negative linear correlation with temperature (Figure 3a), suggesting that the Langmuir model has the potential to estimate the *n*_L_ at different temperatures.

Furthermore, pressure is a key factor affecting the adsorption capacity of gas. As shown in Figure 2, there is a positive correlation between adsorption capacity and pressure. The increase in adsorption capacity with rising pressure can be attributed to higher pressure effectively reducing the binding energy required for gas adsorption and enhancing intermolecular interactions.

In this study, the Langmuir model was utilized to describe this pressure dependence. The Langmuir constant (*K*_L_) is related to the affinity of gas molecules for the adsorbent surface, with larger values indicating a stronger affinity of the gas for the adsorbent. The *K*_L_ values of Mt are considerably higher than those of Sep, indicating H_2_ adsorption occurs more readily on Mt. This also implies that, due to this higher affinity, the adsorbed H_2_ is released less readily from Mt, and the partial pressure must be reduced more strongly to desorb the gas. Moreover, increasing temperature may significantly decrease the H_2_ affinity of clay minerals. As displayed in Table S3, the decrease in *K*_L_ values for both Mt and Sep with increasing temperature suggests higher Langmuir pressure at higher temperatures, leading to a greater desorption capacity. Figure 3b shows a strong linear relationship between the logarithms of the *K*_L_ values of clay minerals and the reciprocal of temperature (1/*T*), aligning with observations in previous studies.^44−46^ As briefly discussed, the effects of varying clay mineral composition and temperature on H_2_ adsorption can be quantified by determining the *K*_L_ at different temperatures.

#### 3.2.3 Thermodynamics of H_2_ Adsorption on Clay Minerals

Adsorption thermodynamics provides crucial information about the heterogeneity of the adsorbent surface. By analyzing the thermodynamics of adsorption, we aimed to understand the mechanisms behind H_2_ adsorption onto clay minerals. The thermodynamics parameters, including enthalpy (Δ*H*) in kJ/mol, entropy (Δ*S*) in J/mol/K, and Gibbs free energy (Δ*G*) in kJ/mol (reported at Table S3), reveal details about the spontaneity and randomness of the adsorption process.

The minus sign of the Δ*H* indicates that the adsorption process is exothermic. The adsorption of H_2_ on Mt (Δ*H =* −15.9) and Sep (Δ*H =* −4.2) is physisorption with weak interactions.^47^ The heat of adsorption for Mt is approximately four times that of Sep, demonstrating stronger interactions between H_2_ and Mt. The negative value of Δ*S* suggests that H_2_ adsorption on the surface of Mt (Δ*S =* −91.2) and Sep (Δ*H =* −54.9) transitions from a random state to an ordered state. Furthermore, the Δ*G* values for both clay minerals are positive, showing that the adsorption process is thermodynamically nonspontaneous under high-pressure conditions. A higher Δ*G* for the absorbent indicates a greater tendency to adsorb gas molecules,^48^ explaining the higher H_2_ adsorption capacity on Sep. Additionally, the Δ*G* values increase with temperature, reflecting a decline in adsorption feasibility at higher temperatures. This is consistent with the exothermic nature of the adsorption process, indicating that high temperatures are not conducive to adsorption.

#### 3.2.4 H_2_ Adsorption Capacity Estimation Algorithms and Adsorption Capacity as a Function of Depth

H_2_ adsorption in geological conditions can be preliminarily estimated based on adsorption parameters derived from experimental results, as demonstrated by previous researches.^23^ Here, it is assumed that the thermodynamic parameters for H_2_ adsorption on dry samples might apply to actual reservoir conditions with high temperatures.

The adsorption capacity *n*(*z*) as a function of depth is influenced by both the corresponding temperature *T*(*z*) and the hydrostatic pressure profile *P*(*z*) at a specific location within the reservoir. The following equations describe this relationship
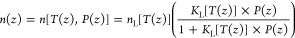
5
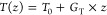
6
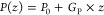
7Where *T*(*z*) (K) and *P*(*z*) (MPa) represent adsorption temperature and pressure at depth *z*, respectively; *T*_0_ (K) and *P*_0_ (MPa) indicate land surface temperature and pressure, respectively. *G*_T_ (K/km) and *G*_P_ (MPa/km) denote the geothermal and hydrostatic pressure gradients, respectively, while *z* (m) is burial depth.

Linear regression analysis yielded the following relationship for *n*_L_ and *K*_L_
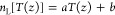
8
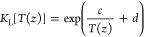
9Where the coefficients *a*, *b*, *c*, and *d* were determined using the linear regression method. Table S4 summarizes the adsorption parameters used in this study.

**Figure 4:**
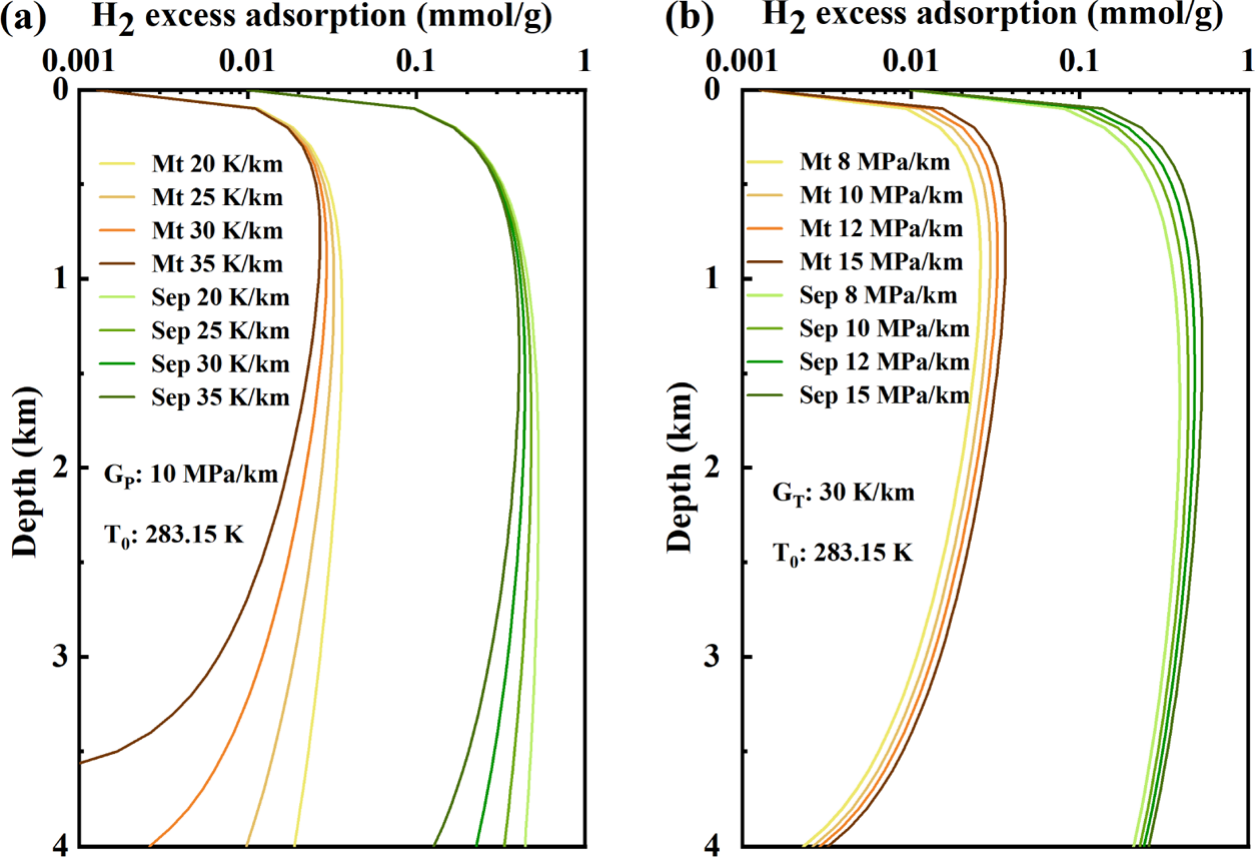
(a) Variations in adsorption capacities with burial depths corresponding to different geothermal gradients. (b) Variations in adsorption capacities with burial depths corresponding to different pressure coefficients.

Figure 4 illustrates the relationship between the gas adsorption capacities and varying sampling depths, considering adsorption property curves at a surface temperature of 283.15 K, and different *G*_T_ and *G*_P_. The adsorption capacity at different *G*_T_ and *G*_P_ values gradually increases with burial depth, reaching a peak before declining. At the same depth, the adsorption capacity decreases with increasing *G*_T_. Additionally, differences in adsorption capacity at different *G*_T_ values become more pronounced with increasing depth (Figure 4a), indicating more significant effects of *G*_T_ on adsorption potential at greater burial depths. A higher *G*_T_ results in a steeper decline rate of adsorption capacity. In comparison to Sep, the H_2_ adsorption of Mt exhibits a more sensitive response to temperature. Conversely, the adsorption capacity increases with respect to *G*_P_, and overpressure reservoirs exhibit higher adsorption capacity. As burial depth increases, differences in adsorption capacity at different *G*_P_ values decrease (Figure 4b). Above 1300 m, the effect of pressure on the H_2_ adsorption capacity of Sep is greater than that of Mt. Below 1300 m, the H_2_ adsorption capacity of Mt exhibits a more sensitive response to pressure. There is a turning point in depth above which pressure predominantly influences adsorption capacity (shallow burial stage), while temperature becomes the dominant factor at greater depths (deep burial stage). The corresponding turning points for Mt and Sep in this study were 1050 and 1750 m, respectively. In summary, reservoir adsorption capacity results from the coupling of the positive effect of reservoir pressure and the adverse effect of the reservoir temperature. Consequently, low-temperature overpressure reservoirs are more conducive to gas storage through adsorption.

However, it should be noted that predicting adsorption capacity by extrapolating reservoir temperature and pressure conditions is a complex process. Adsorption amounts measured on dry samples may not accurately reflect actual reservoir conditions and should be considered a maximum estimate. Additionally, the presence of brine in actual reservoirs can affect adsorption by changing mineral surface properties (like wettability) and the ionic strength of the medium.^49^ In addition, the presence of organic acids can alter the wettability of the reservoir, thereby affecting gas capture and the accurate calculation of reservoir capacity.^50,51^ Thus, future research should undertake a comprehensive assessment of multiple factors, including reservoir composition, the presence of cushion gases, the impact of moisture, mineralogical heterogeneity, pressure cycling, surface chemistry, gas diffusion rates, and the role of organic matter. This approach will yield a more complete understanding of H_2_ interactions within subsurface geological reservoirs.

#### 3.2.5 Excess Adsorption and Desorption: Assessing Hysteresis and Injection/Production Cycles

The operational flexibility required for frequent H_2_ injection and extraction in geological H_2_ storage underscores the need to thoroughly understand H_2_ adsorption–desorption dynamics. This understanding is essential for an accurate evaluation of storage stability and for quantifying potential losses in H_2_ recovery.

**Figure 5:**
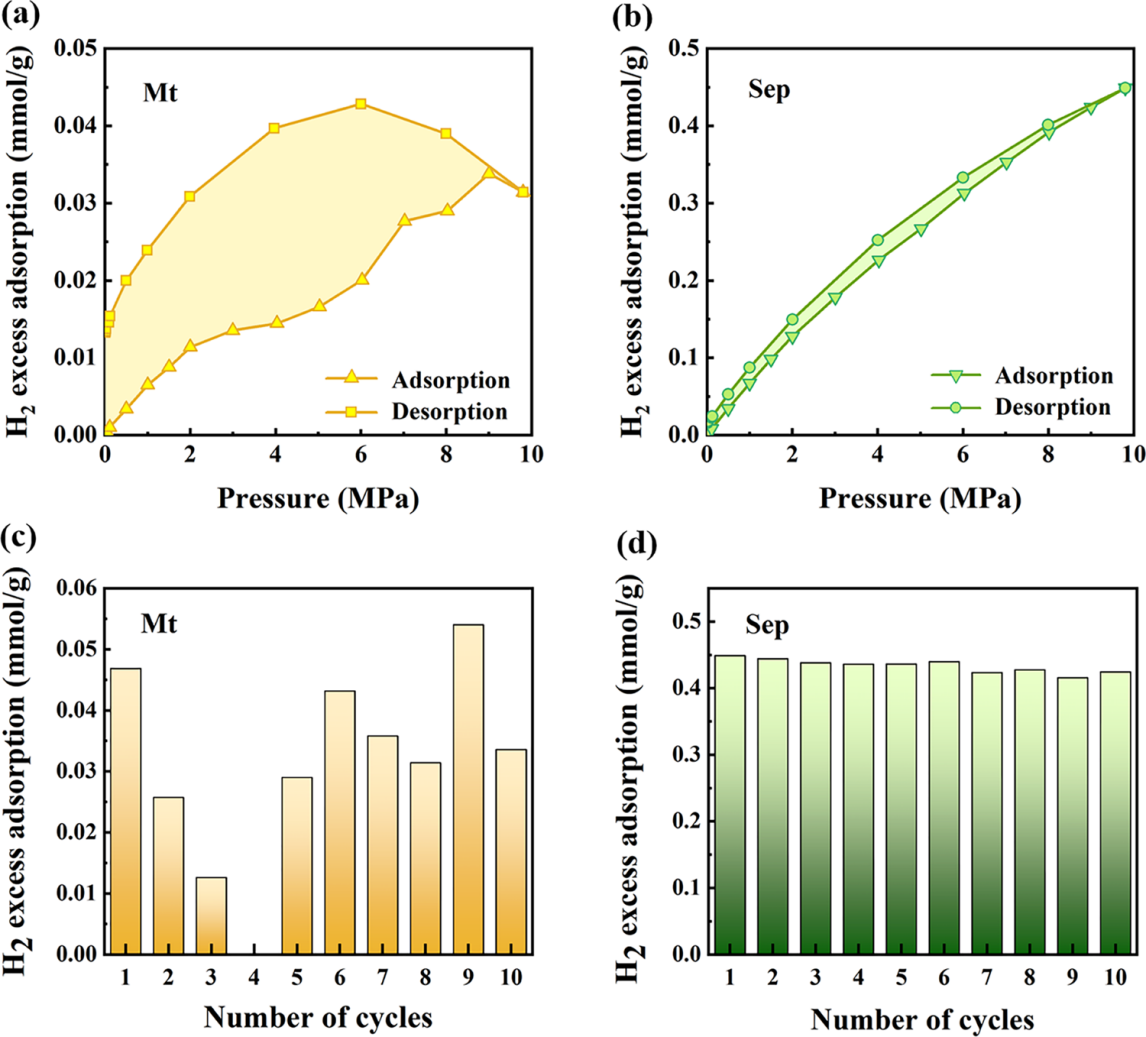
H_2_ adsorption and desorption properties of clay minerals at 313.15 K: (a) H_2_ adsorption and desorption isotherms of Mt, (b) H_2_ adsorption and desorption isotherms of Sep, (c) The impact of pressure cycling on H_2_ adsorption capacities of Mt, (d) The impact of pressure cycling on H_2_ adsorption capacities of Sep.

In our findings, montmorillonite exhibited pronounced deviations between adsorption and desorption curves, indicative of strong hysteresis. This behavior points to a potentially incomplete recovery of the adsorbed H_2_, as illustrated in Figure 5a. The estimated loss of H_2_ for Mt stood at approximately 42.19%. In contrast, sepiolite demonstrates considerably less hysteresis, as shown in Figure 5b, with an H_2_ loss rate of only about 3.56%. This suggests a higher ease of access to the adsorbed H_2_ molecules within Sep.

We observed two distinct types of desorption isotherms. Mt was characterized by no decline in adsorption capacity during the initial desorption process and even showed an increase in adsorption capacity at the beginning. On the other hand, Sep exhibited an immediate decline in the amount of adsorbed gas with as the gas pressure decreased.

The observation corroborates the findings presented in Section 3.2.2, concerning the Langmuir equation fitting for *K*_L_, reaffirming the applicability of the Langmuir model for H_2_ adsorption on clay minerals. Furthermore, the degree of hysteresis can be quantitatively characterized by a hysteresis index (*HI*)
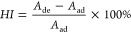
10Where *A*_ad_ and *A*_de_ are the areas under the isothermal adsorption and desorption curves, the *HI* of Mt was significantly higher than that of Sep, with values of 89.93 and 7.11%, respectively.

Hysteresis has been widely observed in the adsorption of CH_4_ and CO_2_ on coal and shale; however, the underlying causes of this phenomenon remain unresolved.^52−55^ In evaluating the hypotheses proffered by various researchers, and in the context of our experimental conditions, we believe that adsorption-induced pore structure alterations could be a contributing factor to the observed hysteresis.^55,56^ The presence of nanopores within clay minerals allows for the tightly bound embedding of gas molecules under high pressure, potentially leading to pore deformation. These constricted channels can impede the release and diffusion of the adsorbed gas, giving rise to hysteresis.

The intensified hysteresis observed in Mt may be attributed to its tendency to swell,^17,52,57^ a characteristic which likely amplifies the pore structure modifications and results in reduced pore connectivity upon desorption as opposed to adsorption. Additionally, in Sep the micropores are more uniformly interconnected throughout its fibrous matrix, enhancing the mobility of H_2_ molecules and, thus, potentially reducing hysteresis.

Hysteresis in adsorption and desorption cycles affects different gases differently. In the context of CO_2_ storage, hysteresis may play a beneficial role by stabilizing the trapped gas and thus enhancing sequestration effectiveness.^54^ This stabilization can be advantageous for containing highly diffusive gases like H_2_, potentially mitigating leakage. However, from an energy extraction perspective, hysteresis could signify a disadvantage for both H_2_ and CH_4_. This is because it introduces a degree of uncertainty into reliably estimating the amount of recoverable and producible reserves. It could also affect the stability and predictability of gas production over time.

To assess the impact of pressure cycling on the H_2_ adsorption capacities of montmorillonite and sepiolite, experiments were conducted at a constant temperature of 313.15 K. Figure 5c,d present the H_2_ adsorption at a pressure of 10 MPa.

For Mt, the data did not exhibit a discernible trend across the pressure cycles. This absence of a specific pattern could likely be attributed to the hysteresis effect observed during the adsorption–desorption tests, as it indicates that the adsorption behavior may not be entirely reversible under these conditions.

In contrast, Sep showed consistent H_2_ adsorption measurements for each cycle, suggesting minimal or no hysteresis influence within this material. This consistency across cycling indicates that Sep may present more predictable and stable adsorption properties under repeated pressure variations.

### 3.3 Adsorption Capacities for H_2_, N_2_, CH_4_, and CO_2_ on Clay Minerals

**Figure 6:**
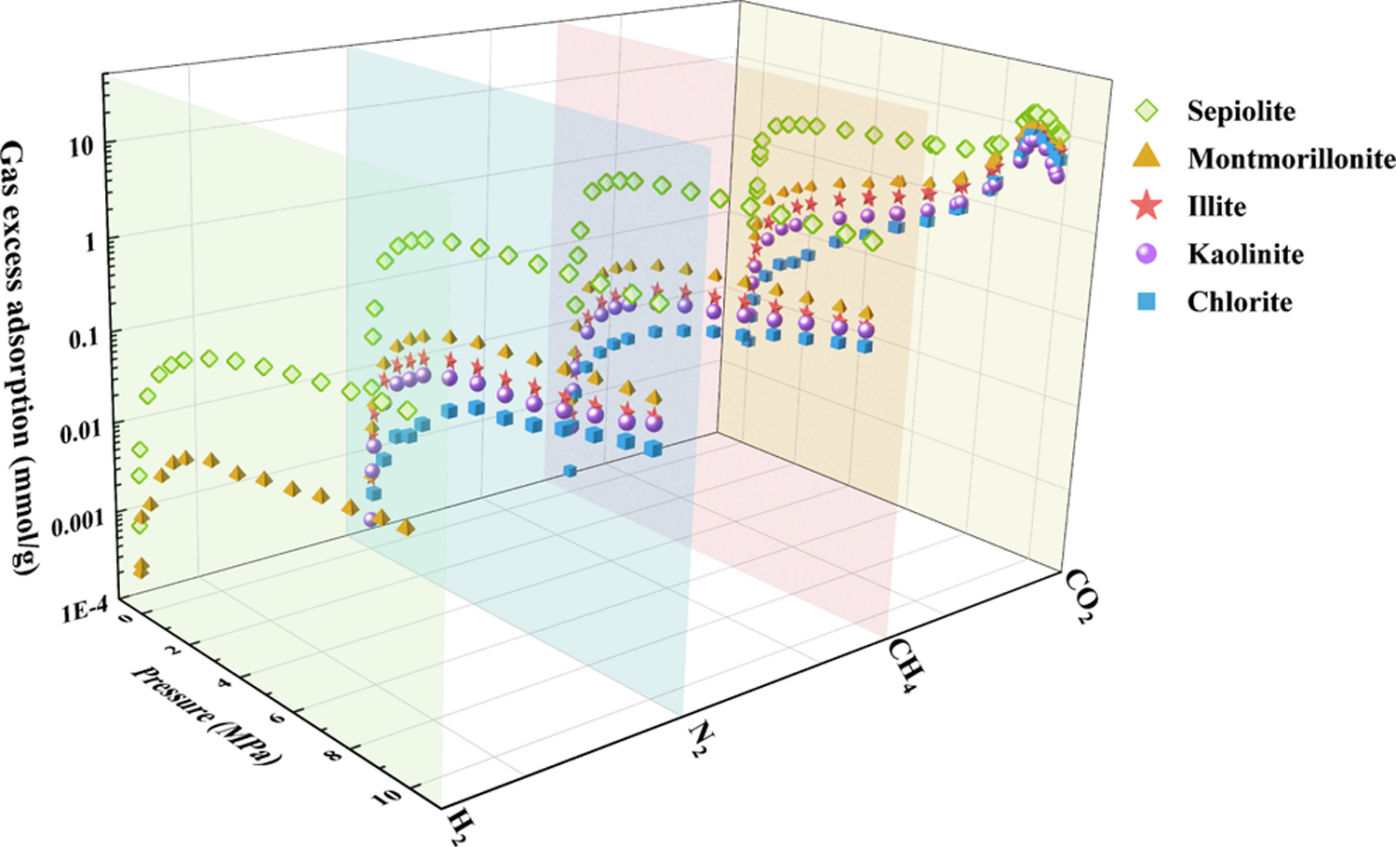
Adsorption isotherm of H_2_ and cushion gases (CO_2_, CH_4_, and N_2_) on clay minerals up to 10 MPa and 313.15 K.

Understanding the adsorption capacities of clay minerals for key gases such as hydrogen (H_2_) and potential cushion gases is fundamental to developing effective hydrogen storage strategies. To this end, we have evaluated and compared the adsorptive properties of these minerals concerning CO_2_, CH_4_, and N_2_. The adsorption capacity for each gas was quantitatively determined and is presented in Figure 6.

We observed a direct correlation between gas adsorption capacities and the specific surface area along with micropore volume among the clay minerals studied. The gas uptakes follow this order: Sep ≫ Mt > Il > Kaol > Chl (Table 1, Figure 6). The reason for this trend can be explained by the intrinsic properties of micropores. Micropores, being of a small size, facilitate a strong binding capacity for gas molecules. Additionally, micropores substantially contribute to the total specific surface area, providing more adsorption sites and significantly affecting gas adsorption capacity. Considering the distinctive microporous structures, it is evident from our experimental results that micropores play a pivotal role in the adsorption process for clay minerals. Hence, analyzing the pore structure holistically, we conclude that microporosity is the principal determinant in the gas adsorption behavior of the clay minerals evaluated in this study.

The adsorption capacity of clay minerals is inherently connected to their pore structure and the properties of the adsorbed gas. In our study, we observed a hierarchy in the adsorption capacity for varying gases on the clay sample, following the order: CO_2_ ≫ CH_4_ > N_2_ > H_2_. This order mirrors differences in molecular characteristics that influence adsorption behavior.

CO_2_ possesses a strong quadrupole moment, which enhances its interaction with the negatively charged surfaces of clay minerals (see Table S5). This strong electrostatic attraction results in a higher adsorption capacity for CO_2_. In contrast, CH_4_ has a zero quadrupole moment, and thus, the discrepancy in adsorption capacities between CH_4_ and N_2_ cannot be solely explained by their quadrupole moments.

Molecular size and structure also play a significant role in adsorption behavior. CO_2_’s smaller kinetic diameter and linear structure, compared to CH_4_ and N_2_, facilitate its diffusion within constrained pore structures. Moreover, the abundance of oxygen-containing functional groups in the tested clays (see Figure 1e) can form additional interactions with CO_2_, further increasing its adsorption capacity.^58^

The adsorption isotherm of CO_2_ is distinctive, reflecting a significant density change associated with its phase transition. This transition is characterized by three stages: a slow increase, a rapid increase, and a sudden decrease (see Figure 6). Upon reaching the supercritical state, the disparities in adsorption capacity between clay minerals diminish, while the overall magnitude of adsorption capacity undergoes alterations. This phenomenon promotes CO_2_-clay interaction by enhancing binding energy, resulting in the attachment of more molecular layers onto the surfaces.^59^

Polarizability is another critical factor affecting adsorption behavior. The order of polarizabilities of the tested gases in our study is as follows: CO_2_ > CH_4_ > N_2_ > H_2_^60^ (see Table S5). The adsorption capacities of gases on clay minerals at 313.15 K follow the order of their polarizability rather than their quadrupole moment values. Polarizability affects the extent of induced dipole interactions, which can explain the variations in adsorption capacities for CH_4_, N_2_, and H_2._^61−63^

Lastly, the interaction energy with the adsorbent surfaces significantly impacts adsorption behavior. H_2_, with its low polarizability and weaker quadrupole moment, primarily engages through weak van der Waals forces with the clay mineral surfaces,^21,33^ which are not as strong as the interactions observed with other gases. Similar phenomena have been reported in gas adsorption studies involving metal–organic frameworks,^64^ underground coal seams,^21,65^ and organic-rich source rocks.^33^

## 4 Implications for Target Reservoir and Cushion Gas Selection

The systematic investigation of H_2_ adsorption on a variety of clay minerals commonly found in reservoirs is instrumental for informing the selection of sites for large-scale geological H_2_ storage. Among the minerals tested, sepiolite demonstrated a superior adsorption capacity, highlighting its potential for enhanced H_2_ storage efficiency in geological formations.

Montmorillonite exhibited pronounced adsorption hysteresis, which could hinder the effective recovery of stored hydrogen and complicate the estimation of accurate adsorption capacities. Additionally, its swelling tendency raises concerns about the structural integrity of the reservoirs, as such changes might lead to compromised containment and introduce pathways for leaks.

Illite, chlorite, and kaolinite were found to have negligible H_2_ adsorption at temperatures typical of reservoir conditions, suggesting a limited role in conventional geological H_2_ storage scenarios.

Therefore, from a purely mineralogical perspective, reservoirs that are rich in sepiolite might be more appropriate for H_2_ storage applications compared to those with a higher content of montmorillonite or other low-adsorbing clays. However, the selection of suitable reservoirs should not be based solely on clay mineral composition. A comprehensive assessment incorporating geotechnical evaluations, caprock integrity, economic feasibility, and long-term operational considerations must also be conducted to determine the overall appropriateness of any given geological formation for H_2_ storage.

In terms of cushion gas selection for enhancing the efficiency of geological storage systems, our comparative adsorption experiments highlighted CO_2_’s superior adsorption capacity among various gases. This characteristic indicates that utilizing CO_2_ as a cushion gas could increase the overall storage capacity of the reservoir while concurrently facilitating carbon sequestration, which is an additional environmental benefit.

However, practical considerations must temper our selection. Although Illite, chlorite, and kaolinite have shown the capability to adsorb N_2_, CH_4_, and CO_2_ at reservoir temperatures, each gas’s impact on reservoir and containment system stability varies. For instance, the swelling effect of montmorillonite in the presence of CO_2_ can reduce caprock permeability, aiding in the containment of stored gases.^11^ However, when interacting with supercritical CO_2_, certain caprock minerals may undergo dissolution, which can impact the long-term integrity of the caprock by increasing its porosity and permeability. This, in turn, might create leakage pathways for the stored CO_2_. On the other hand, the precipitation of carbonate minerals can have a beneficial effect by potentially enhancing the caprock’s sealing capacity through the natural plugging of pores and fractures. Therefore, it is vital to understand the specific geochemical interactions within the caprock, which can either be induced by the exposure to CO_2_ or influenced by its phase state.

Therefore, while from an adsorption standpoint CO_2_ appears to be an advantageous choice, the selection of a cushion gas should not be solely dictated by adsorption capacity. It must entail a holistic appraisal encompassing geochemical interactions, the mechanical behavior of the host formations, long-term storage integrity, environmental outcomes, and economic efficacy. Only through such a multidisciplinary evaluation can an informed decision be made that aligns storage goals with sustainability and safety considerations.

Finally, when comparing adsorption results across different studies, it is critical to account for variations in clay samples (e.g., purity, pore structure), experimental methods (e.g., measurement techniques, choice of equation of state, degassing conditions, fitting methods), and error calibration procedures (e.g., dead volume, blank corrections, reproducibility). These factors can lead to discrepancies between results; thus, comparisons should be made with caution, ensuring that similar standards and techniques are being evaluated to obtain a more accurate assessment.

## 5 Conclusions

In this study, we systematically evaluated the adsorption performance of various clay minerals commonly found in geological formations, including montmorillonite (Mt), Illite (Il), chlorite (Chl), sepiolite (Sep), and kaolinite (Kaol), for H_2_ and potential cushion gases (N_2_, CH_4_, and CO_2_). Our findings highlight several key insights into the potential application of these minerals in geological hydrogen storage.

Among the tested clay minerals, sepiolite exhibited the highest H_2_ adsorption capacity, indicating its suitability as a favorable component in geological formations for hydrogen storage. The Langmuir monolayer theory effectively describes the interaction of hydrogen adsorption on these clay minerals, revealing that the adsorption process is characterized by physisorption, is nonspontaneous, and exothermic.

We developed a comprehensive model based on Langmuir theory to assess the hydrogen adsorption capacity of montmorillonite and sepiolite under varying pressure gradients, temperature gradients, and depths. The results show that montmorillonite’s hydrogen adsorption capacity is more sensitive to temperature variations compared to sepiolite. Notably, there is a critical depth level beyond which temperature becomes the dominant factor influencing adsorption capacity over pressure. Consequently, low-temperature, overpressure reservoirs are more favorable for gas storage via adsorption.

The study also uncovered significant differences in adsorption hysteresis between the minerals. Montmorillonite exhibits severe adsorption hysteresis, resulting in a hydrogen loss of approximately 42.19%, whereas sepiolite shows relatively weaker hysteresis, with a loss of only 3.56%. This hysteresis is likely due to changes in pore structure induced by the adsorption and desorption processes, affecting the accessibility of gas molecules. The strong hysteresis in montmorillonite may be attributed to its tendency to swell upon gas adsorption.

The gas adsorption capacity hierarchy observed in this study is sepiolite > montmorillonite > Illite > kaolinite > chlorite, with CO_2_ exhibiting the highest adsorption capacity among the gases, followed by CH_4_, N_2_, and H_2_. The adsorption performance of these clay minerals is closely linked to their pore structure, including surface area and micropore volume, as well as the physical properties of the gases.

From an adsorption perspective, using CO_2_ as a cushion gas appears advantageous, as it not only enhances hydrogen storage capacity but also facilitates carbon sequestration. However, this potential benefit must be carefully weighed against a comprehensive understanding of geological, environmental, and economic implications to ensure the overall viability and sustainability of the storage operation.

Our study provides crucial insights into the adsorption properties of clay minerals and their interaction with hydrogen. Understanding these dynamics is essential for understanding the effectiveness of caprocks in geological hydrogen storage. Our findings contribute to the broader effort of developing efficient and sustainable hydrogen storage solutions. These insights are important for advancing geological hydrogen storage technologies and natural hydrogen exploration, playing a major role in the transition toward a sustainable energy future.

## Supporting Information Available

Experimental conditions, the results of XRF measurements, Langmuir model parameters, thermodynamic properties of adsorption, the parameters for estimating adsorption capacity as a function of depth, and the kinetic diameter, the quadrupole moment, and the polarizability of the adsorbate gases (PDF)
