# Supplementary material for: Hydrogen and Cushion Gas Adsorption–Desorption Dynamics on Clay Minerals
Source: ACS Appl Mater Interfaces. 2024 Sep 26;16(40):53994–4006. doi: 10.1021/acsami.4c12931 (PMC11472264; doi:10.1021/acsami.4c12931)
Supplement: Supplementary file 1 — am4c12931_si_001.pdf [file am4c12931_si_001.pdf]

Supporting Information

Hydrogen and Cushion Gases Adsorption-Desorption Dynamics on Clay Minerals

Qian Zhang [a], Mohammad Masoudi [b,c], Lingjie Sun [a], Lunxiang Zhang [a,d*], Lei Yang [a,d], Yongchen Song [a,d], Aliakbar Hassanpouryouzband [e*]

a) Key Laboratory of Ocean Energy Utilization and Energy Conservation of the Ministry of Education, Dalian University of Technology, Dalian 116024, China
b) Department of Geosciences, University of Oslo, P.O. Box 1047 Blindern, 0316 Oslo, Norway
c) SINTEF Industry, Applied Geoscience Department, 7465 Trondheim, Norway
d) Ningbo Institute of Dalian University of Technology, No.26 Yucai Road, Jiangbei District, Ningbo 10315016, China
e) School of Geosciences, University of Edinburgh, Grant Institute, West Main Road, Edinburgh EH9 3FE, United Kingdom

This document presents 5 Tables.

Table S1 provides a summary of the experimental conditions. Table S2 outlines the elemental composition of clay samples from XRF. Table S3 gives an overview of Langmuir model parameters and thermodynamic properties of adsorption. Table S4 summarizes the parameters for estimating adsorption capacity as a function of depth. Table S5 presents the kinetic diameter, the quadrupole moment, and the polarizability of the adsorbate gases.

[*] Corresponding Authors: L.Z: lunxiangzhang@dlut.edu.cn, A.H: Hssnpr@ed.ac.uk

Experimental conditions

The experimental conditions of this study encompassed a pressure range from 0 to 10 MPa, with specific experimental temperatures and adsorbed gases detailed in Table S1.

Table S1. Description of the experimental conditions of this study.

**Sample**	**Experimental temperature (K)**					
	273.15	283.15	293.15	303.15	313.15	323.15
**Mt**	H_2	H_2	H_2	H_2	H_2, N_2, CH_4, CO_2	H_2
**Il**	H_2	H_2			H_2, N_2, CH_4, CO_2	
**Chl**	H_2	H_2			H_2, N_2, CH_4, CO_2	
**Sep**	H_2	H_2	H_2	H_2	H_2, N_2, CH_4, CO_2	H_2
**Kaol**	H_2	H_2			H_2, N_2, CH_4, CO_2	

Characterization of clay minerals

To thoroughly characterize the chemical composition of the tested pure clay samples, we employed X-ray fluorescence (XRF) spectroscopy, the results of which are presented in Table S2.

Table S2. Composition of clay samples based on XRF (wt.%).

	**Mt**	**Il**	**Chl**	**Sep**	**Kaol**
**SiO_2**	66.00	52.06	31.95	43.32	53.21
**AiO_2**	21.93	32.53	21.51	1.53	44.03
**Fe_2O_3**	2.01	3.39	15.25	22.28	0.77
**MgO**	5.34	1.25	29.26	17.68	
**CaO**	3.24	0.24	0.32	0.25	0.08
**TiO_2**	0.28	0.23	1.03	0.06	0.30
**K_2O**	0.42	9.84		0.39	0.52
**ZnO**	0.12	0.29	0.23	0.14	0.07
**P_2O_5**		0.13	0.08	0.07	0.51
**MnO**	0.09		0.13	0.50	
**Na_2O**	0.25			0.50	0.21

Langmuir model parameters and thermodynamic properties of adsorption

We used Langmuir model to analyse our experimental H_2 adsorption isotherms. The details of the model's parameters are reported in Table S3.

The thermodynamics parameters, including enthalpy (ΔH) in kJ/mol, entropy (ΔS) in J/mol/K, and Gibbs free energy (ΔG) in kJ/mol are also reported at Table S3.

Table S3. Results of Langmuir parameters and thermodynamic properties of adsorption on selected samples across various temperature conditions.

**Samples**	**Experimental conditions (K)**	n_L **(mmol/g)**	K **(1/MPa)**	P_L **(MPa)**	R^2	ΔH **(kJ/mol)**	ΔS **(J/mol/K)**	ΔG **(kJ/mol)**
**Mt**	273.15	0.0849	0.2439	4.1000	0.9886	-15.9	-91.2	3.2
	283.15	0.1002	0.1197	8.3542	0.9985			5.0
	293.15	0.0870	0.0974	10.2669	0.9764			5.7
	303.15	0.0713	0.0958	10.4384	0.9915			5.9
	313.15	0.0679	0.0850	11.7647	0.9847			6.4
	323.15	0.0607	0.0675	14.8148	0.9534			7.2
**Sep**	273.15	1.3137	0.0840	11.9048	0.9999	-4.2	-54.9	5.6
	283.15	1.2275	0.0817	12.2414	1.0000			5.9
	293.15	1.2055	0.0783	12.7796	0.9984			6.2
	303.15	1.0731	0.0740	13.5062	1.0000			6.6
	313.15	1.0123	0.0669	14.9477	0.9965			7.0
	323.15	0.9534	0.0638	15.640	0.9999			7.4

Parameters for estimating adsorption capacity as a function of depth

The coefficients a, b, c, and d in Equations 8 and 9 in the manuscript were determined using the linear regression method and are summarized in Table S4.

Table S4. The parameters for investigating adsorption capacity as a function of depth.

	**Mt**	**Sep**
**a**	-0.0007	-0.0074
**b**	0.2777	3.3283
**c**	1913.52	507.55
**d**	-8.6673	-4.3062
$\mathbf{T_0}$	283.15 K	
$\mathbf{G_T}$	20, 25, 30, 35, 40 K/km	
$\mathbf{G_P}$	8, 10, 12, 15, 20 MPa/km	

Properties of the adsorbate gases

Properties of the adsorbate gases, including the kinetic diameter, the quadrupole moment, and the polarizability is summarised in Table S5.

Table S5. Properties of the adsorbate gases, including the kinetic diameter, the quadrupole moment, and the polarizability[1,2].

Adsorbate gas	Kinetic diameter (Å)	Quadrupole moment ($\times 10^{40}$ cm^2)	Polarizability ($\times 10^{-25}$ cm^3)
CO_2	3.3	-13.71	29.1
CH_4	3.8	0.00	25.9
N_2	3.64	-4.91	17.4
H_2	2.89	2.2	8.1

References

(1) Beda, A.; Vaulot, C.; Ghimbeu, C. M. Hard Carbon Porosity Revealed by the Adsorption of Multiple Gas Probe Molecules (N2, Ar, CO2, O2 and H2). *J. Mater. Chem. A* **2021**, *9* (2), 937–943. https://doi.org/10.1039/d0ta10088a.

(2) Rallapalli, P.; Prasanth, K. P.; Patil, D.; Somani, R. S.; Jasra, R. V; Bajaj, H. C. Sorption Studies of CO2, CH4, N2, CO, O2 and Ar on Nanoporous Aluminum Terephthalate MIL-53(Al). *J. Porous Mater.* **2011**, *18* (2), 205–210. https://doi.org/10.1007/s10934-010-9371-7.
